# Death of Professor Himly

**Published:** 1837-07-01

**Authors:** 


### 3. Death of Professor Himly.

The celebrated chemist, Professor Himly, of Goettingen, was drowned on the 15th ult. in the most extraordinary manner. He was taking his morning's walk, and being absorbed in reflection, it is supposed, forgot his proximity to the river Leine, fell into it, and perished before any assistance could reach him. His loss to the scientific world will be irreparable.—*Times.*

